# The Antiemetic Mechanisms of Gingerols against Chemotherapy-Induced Nausea and Vomiting

**DOI:** 10.1155/2022/1753430

**Published:** 2022-02-24

**Authors:** Yongzhao Dai, Yaozhong Zhao, Ke Nie

**Affiliations:** School of Chinese Materia Medica, Guangdong Pharmaceutical University, Guangzhou 510006, China

## Abstract

Chemotherapy-induced nausea and vomiting (CINV) is a common and painful side effect that occurs in cancer patients receiving chemotherapeutic drugs. Although an abundance of agents are applied to prevent CINV, there is still lack of effective control in delayed nausea and vomiting. Ginger (*Zingiber officinale* Rosc.), a traditional antiemetic herb, draws attention due to its therapeutic effect in treating acute and delayed CINV. Its main bioactive pungent constituents, gingerols, contribute to the antiemetic effect against CINV primarily. A growing number of reports have made progress in investigating the mechanisms of gingerols and their single ingredients against CINV. In this review, we searched for relevant studies in PubMed database to summarize the mechanism of gingerols in the prevention of CINV and provided a preliminary prediction on the potential targets and signaling pathways using network pharmacology, laying a foundation for further researches.

## 1. Introduction 

Chemotherapy-induced nausea and vomiting (CINV) is a side effect that occurs in antineoplastic chemotherapies and severely affects the compliance as well as life quality of cancer patients [1]. The underlying mechanisms of CINV have not been fully clarified yet. The major mechanism of CINV is concerned with the alteration of neurotransmitters in central and peripheral, such as 5-hydroxytryptamine (5-HT), substance P (SP), and dopamine (DA) [2]. Through binding with 5-HT type 3 receptor (5-HT_3_R) and neurokinin-1 receptor (NK-1R), 5-HT and SP are closely related to the onset of acute phase and delayed phase of CINV, respectively. The 5-HT_3_R antagonist like ondansetron and NK-1R antagonist like aprepitant are the basic clinical prophylaxis to treat CINV [3, 4]. Although the antiemetic effect of these antagonists seems promising, adverse effects like headache, constipation, and fatigue commonly occur [2]. Besides, 5-HT_3_R antagonist alone is less effective in relieving delayed CINV, while combining with the NK-1R antagonist is effective in treating delayed emesis, it calls for large medical cost to patients [2]. Therefore, CINV remains as a great restriction for the usage of chemotherapy agents in clinical cancer treatments. There is an urgent need for further investigating the mechanism of CINV, as well as exploring novel medicines with less side effects and promising antiemetic property in controlling delayed nausea and vomiting.

Ginger (*Zingiber officinale* Rosc.), a traditional and common herb in Asia and Europe, has been used as a vital approach in mitigating nausea and vomiting for more than 2000 years [5]. Clinical trial had proven the antiemetic effect of ginger against acute and delayed phases of CINV. Pillai et al. [6] and Uthaipaisanwong et al. [7] indicated that ginger capsules were effective in acute and/or delayed phase of CINV and that ginger could be an additional therapy to standard nausea and vomiting prophylaxis protocol. Also, oral intake of ginger or given ginger with high-protein meals markedly reduced delayed nausea and vomiting [8, 9]. Preclinical studies indicated that the inhibition of 5-HT_3_R largely contributed to the antiemetic effect of ginger, which largely depends on its pharmacological active constituent gingerols [10, 11]. Gingerols, consisting of various structural analogs including 6-, 8-, 10-gingerol and 6-, 8-, 10-shogaol, are the major pungent constituents and fraction of ginger [12]. Konmun et al. conducted a phase II clinical study and showed that 6-gingerol significantly reduced CINV in patients receiving highly emetogenic chemotherapy [13]. And there are a growing number of reports that have made progress in revealing the underlying mechanism of gingerols against CINV in animal models [14, 15].

Up to date, only 5 manuscripts are searched out when using the terms “gingerols or 6-gingerol” and “CINV” in PubMed. Among these studies, there are one review on the mechanisms of ginger against CINV, one clinical trial on the effect of 6-gingerol against chemotherapy-induced emesis in cancer patients, one mechanism study of gingerols on cisplatin-induced emesis, and two in silico studies. However, other studies that investigated the antiemetic mechanism of ginger also involved the antiemetic mechanism of gingerols or its monomers, which have not been systemically summarized. By using the terms “ginger”, “gingerols”, “6-gingerol”, “8-gingerol”, “10-gingerol”, “6-shogaol”, “8-shogaol”, “10-shogaol” and “chemotherapy”, “cisplatin”, “5-HT”, “SP”, “DA”, and “gastrointestinal”, we searched for studies in PubMed database from inception until Nov 13, 2021, based on the following criteria and consistencies: (1) The keywords include ginger and/or *Zingiber officinale* Rosc., gingerols, shogaols, 6-, 8-, 10-gingerol, 6-, 8-, 10-shogaol, chemotherapy, and nausea and/or vomiting. (2) The clinical trials of gingerols or its monomers against CINV. (3) The mechanism studies of gingerols or its monomers against CINV, especially on the mediation of 5-HT, SP, DA signaling pathways, and gastrointestinal function. In this review, we summarized the mechanism studies of gingerols in treating CINV and used network pharmacology to predict potential targets and pathways, providing new prospects on the basic of previous investigations.

## 2. The Pathological Mechanisms of CINV

Depending on the occurrence time of nausea and sickness after chemotherapy, CINV is classified into 5 types: acute, delayed, anticipatory, breakthrough, and refractory [16]. Acute CINV usually occurs minutes or hours after chemotherapy and reaches the peak at 5–6 hours, mainly concerns with 5-HT in central and gastrointestinal tract. Delayed CINV often occurs 24 hours after chemotherapy and reaches the peak at 72 hours and is primarily mediated by SP in central. Anticipatory CINV refers to the nausea and vomiting due to the anxiety and tension before next chemotherapy, for the poor control of sickness occurred in the previous chemotherapy. Breakthrough CINV is the sickness in spite of proper prophylaxis after chemotherapy, and refractory CINV happens following breakthrough CINV in the subsequent chemotherapy cycles. Both breakthrough and refractory CINV result in nausea and vomiting in response to the latest chemotherapeutic treatment [17, 18].

The mechanisms of CINV have not been fully understood; it has been reported to interact between central nervous system and gastrointestinal tract mediated by neurotransmitters, like 5-HT and SP [19]. Chemotherapeutic agents damage intestinal mucosa through oxidative stimulation and via irritating enterochromaffin (EC) cells to release 5-HT. And 5-HT combines with 5-HT_3_R; then, the vagal afferent depolarizes and transmits nervous impulse to the vomiting center (VC), triggering vomiting behaviors. Besides, chemotherapy drugs also directly cause emesis via upregulating SP level and increasing the expression of NK-1R in the chemoreceptor trigger zone (CTZ) and VC [20]. Therefore, current CINV prophylaxes are mostly concerned with the blockage of neurotransmitters from binding to corresponding receptors.

## 3. The Major Bioactive Constituents of Gingerols

The bioactive compounds in ginger are varied [12, 21]. Li et al. established a qualitive analysis to reveal the phytochemical constituents of ginger rhizomes extract, and the compounds were mainly characteristic as diarylheptanoids, gingerols, and others [22]. Gingerols are the main pungent constituents and important nutraceutical principles of ginger and can be divided into different constituents based on the different chains connected with the functional group, such as gingerols, shogaols, zingerones, gingerdiones, and gingerdiones [21].

**Figure 1:**
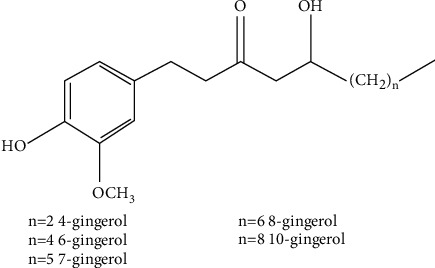
The structure of gingerols.

**Figure 2:**
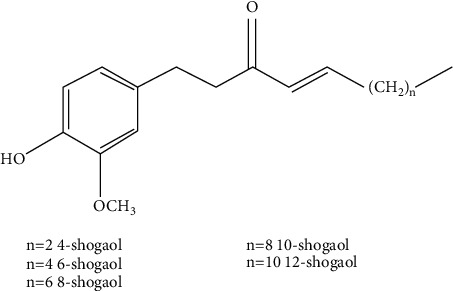
The structure of shogaols.

As a mixture of various analogs, gingerols refer to the ingredients that all contain 3-methoxy-4-hydroxyphenyl functional group [23]. The structure of different monomers in gingerols is formulated based on the amounts of methylene in the unbranched alkyl chains [24]. When the amount of methylene varies from 2, 4, 5, 6 to 8, diverse monomers like 4-, 6-, 7-, 8-, 10-gingerol are composed (Figure 1). For instance, 6-gingerol is formed with the existence of 4 methylenes, whose structure is 1-[4′-hydroxy-3′-methoxyphenyl]-5-hydroxy-3-decanone [25]. According to the Chinese Pharmacopoeia of the People's Republic of China (version 2020), 6-, 8-, 10-gingerol are the quality marker of ginger. In high temperature or under pH 2.5–7.2, gingerols are dehydrated and transformed into shogaols [26]. After eliminating the hydroxide radical at C-5 and formulating a double bond at C-4 and C-5 [27], shogaols are formed from the corresponding gingerols with highly similar structure: 1-[4′-hydroxy-3′-methoxyphenyl]-4-decen-3-one (Figure 2).

The 6-gingerol and 6-shoagol are the representative single ingredients in gingerols [12], which mainly contribute to the antiemetic effect against CINV. Therefore, gingerols, shogaols, and its monomers are primarily concerned in this review.

## 4. The Antiemetic Mechanisms of Gingerols against CINV

Nausea and vomiting can be modeled in species with or without vomiting response. While vomiting can be directly observed in emetic models such as minks, in models like rodents that lack emetic response, the consumption of nonnutritive substances like kaolin clay (i.e., pica behavior) indicates the severity of vomiting [28]. Multiple studies investigated the antiemetic effect of gingerols against CINV in the vomiting model of minks or the pica model of rats induced by chemotherapeutic agents.

### 4.1. Mediating 5-HT Signaling Pathway

5-HT is a monoamine neurotransmitter; about 90% of 5-HT are produced in the intestinal EC cells [29]. Chemotherapy agents stimulate EC cells to release 5-HT, then evoke 5-HT_3_R and transmit stimulus to the brain causing nausea or vomiting [30]. Tryptophan hydroxylase (TPH) is the rate-limiting enzyme that initiates 5-HT synthesis. TPH catalyzes tryptophan to form 5-hydroxytryptophane, with the effect of dehydrogenase, 5-hydroxytryptophane dehydrates and forms 5-HT [31]. Monoamine oxidase A (MAO-A) is the key degrading enzyme of 5-HT, transforming 5-HT into 5-hydroxyindolacetic acid (5-HIAA) [32]. And serotonin reuptake transporter (SERT) controls the reuptake progress of extracellular 5-HT [33]. Therefore, estimating TPH, MAO-A, and SERT levels is crucial in evaluating 5-HT expression.

In the vomiting model of mink, studies had indicated that gingerols, which consisted of analogs with 3-methoxy-4-hydroxyphenyl functional group, significantly ameliorated vomitting behaviour via inhibiting central and peripheral 5-HT systems, suggesting that gingerols significantly ameliorated CINV [14]. Gingerols significantly reduced 5-HT level and 5-HT_3_R expression, which were related to the decrease of TPH that limited 5-HT synthesis, and the increase of SERT that promoted 5-HT degradation in central and peripheral [34]. It is worth noting that gingerols used in the studies mentioned above were purchased from different companies (i.e., Baoji Hongyuan Biotech Co., Ltd. and Xi'an Biotechnology), whose purity and composition proportion were not given. Thus, though the results seem convincing, its reproducibility and reliability are significantly affected. As recorded in the Chinese Pharmacopoeia of the People's Republic of China (version 2020), the total amount of 6-gingerol should not be less than 0.050% and the total amounts of 8-gingerol and 10-gingerol should not be less than 0.040% in ginger. Similarly, the reliability of the studies using gingerols might be improved by defining the total amounts of 6-gingerol, 8-gingerol, 10-gingerol, and other monomers in gingerols.

By isolating pure compounds 6-, 8-, 10-gingerol and 6-shogaol from ginger hexane extract, Abdel-Aziz et al. identified the property of the single ingredients in gingerols on 5-HT systems in N1E-115 cells, isolated rat, and guinea-pig ileum, and equilibrium competition binding studies. It was found that the 5-HT_3_R blocking property of 6-shogaol was the best, followed by 8-shogaol, 8-gingerol, and 10-gingerol, and the effect of 6-gingerol was the second smallest, only greater than 4-gingerol [35, 36]. Besides, the inhibition of pure compound 6-shogaol against emetic signal transmission activated by 5-HT in vagal afferent neurons was better than pure compound 6-gingerol [37]. In vitro study using HEK293 cells and human colon tissue also pointed out that the 5-HT_3_R inhibition of 6-gingerol and 6-shogaol was mainly due to the restriction of 5-HT induced Ca^2+^ influx through 5-HT_3_R [10]. Moreover, a recent study found out that the pure ingredient 6-gingerol ameliorated cisplatin-induced pica and suppressed 5-HT systems in rats. By decreasing the TPH level and increasing the MAO-A, SERT level, 6-gingerol inhibited 5-HT synthesis and facilitated 5-HT metabolism, thereby downregulating 5-HT level as well as inhibiting 5-HT_3_R activation in central and peripheral [15].

In summary, the mechanism of CINV is closely concerned with the activation of 5-HT_3_R, which mediates vomiting behaviors. Gingerols and its single ingredients significantly ameliorate CINV through decreasing 5-HT level and inhibiting 5-HT_3_R expression.

### 4.2. Mediating SP Signaling Pathway

The peptide substance P (SP) presents in area postrema (AP) and nucleus tractus solitarius (NTS). When SP binds to the neurokinin-1 receptor (NK-1R), it results in vomiting [38]. Preprotachykinin-A (PPTA) is the precursor during SP synthesis, and the neprilysin (NEP) is the major tachykinin-degrading enzyme of SP metabolism [39]. Thus, the expression level of PPTA and NEP indicates the anabolic level of SP.

Studies indicated that in the vomiting model of minks and pica model of rats, gingerols significantly ameliorated cisplatin-induced vomiting in mink and kaolin intake in rats through decreasing SP level and inhibiting NK-1R expression in central and peripheral [14, 34, 40]. The inhibition of SP systems is mainly due to the reduction of PPTA, which limited SP synthesis, and the improvement of NEP, which accelerated SP degradation [34]. Similarly, gingerols used in these researches lacked detailed purity and composition proportion, which made the results less convincing. Therefore, further investigation using gingerols with detailed definition on its constituents or pure monomers to study the effect of gingerols on mediating SP system is required.

Taken together, the upregulation of SP and the activation of NK-1R induce CINV. Through reducing PPTA and increasing NEP, gingerols significantly reduce SP level and suppress NK-1R to alleviate CINV.

### 4.3. Mediating DA Signaling Pathway

Besides 5-HT and SP systems, the activation of DA signaling pathway also contributes to CINV. Through dopamine transporter (DAT), DA activates D_2_-like dopamine receptors (D_2_R) that locates in the dorsal vagal complex, central pattern generator, enteric nervous system, gastrointestinal tract, and vagus nerve and then evokes emetic behaviors [41]. Tyrosine hydroxylase (TH) is the rate-limiting enzyme in DA synthesis [42].

In the pica model of rats and vomiting model of minks, studies reported that gingerols significantly ameliorated CINV by inhibiting cisplatin-induced TH increase and DAT reduction and decreasing DA level as well as D_2_R expression in central and peripheral [14, 34, 43]. Likewise, the detailed proportion and purity of gingerols were not given in these studies; further investigation on the effect of gingerols with detailed definition on its constituents or monomers in gingerols on DA system is required.

In summary, the mechanism of CINV is concerned with the activation of DA signaling pathway, and the effect of gingerols against CINV is partly due to the inhibition of DA synthesis, D_2_R activation, and the accumulation of DA metabolism.

### 4.4. Modulating Gastrointestinal Function

Chemotherapeutic treatments not only disturb the level of various neurotransmitters, but also influence gastrointestinal motility. Chemotherapy agents stimulate EC cells to release 5-HT, and the basic physiology of gastrointestinal function depends on 5-HT related signaling pathways [1, 2, 30]. The activation of 5-HT_3_R induced by 5-HT further activates extrinsic nerves and conveys a discomfort signal to the brain to trigger emesis, which could be a potential mechanism of CINV [30]. Besides, chemotherapy agents usually result in delayed gastric emptying. To date, various studies have proven that chemotherapy drug cisplatin significantly reduced gastric emptying and food intake in rats, indicating that the delayed gastric emptying induced by chemotherapy might also be an important factor accounting for CINV [44–46]. Therefore, evaluating the gastrointestinal function after chemotherapy treatment is possible to indicate the severity of CINV.

It was reported that the compound gingerols dose-dependently improved delayed gastric emptying induced by cisplatin and ameliorated chemotherapy-agent induced gastric dysfunctions [43]. Further investigation suggested that pure ingredients 6-, 8-, 10-gingerol and 6-shogaol inhibited 5-HT agonist induced guinea-pig ileum contraction in a dose-dependent manner [36]. And in guinea-pig ileum segment, all these four pure monomers significantly inhibited carbachol response through suppressing cholinergic M3 receptor and 5-HT_3_R [47].

Taken together, the antiemetic effect of gingerols may probably via inhibiting chemotherapy agents induce gastrointestinal dysfunctions.

### 4.5. Others

Apart from interacting with neurotransmitters and modulating gastrointestinal function, chemotherapy agents result in oxidative stress, inflammation, and gastrointestinal dysbacteriosis, which contribute to CINV as well [48–50]. Studies reported that 6-, 8-, 10-gingerol and 6-shogaol exerted antioxidant and anti-inflammation activity, but further investigation is needed to explore the antioxidative effect of gingerols against chemotherapy-induced oxidative stress or inflammation [51, 52]. What is more, 16s rDNA gene analysis of ileum showed that 6-gingerol increased *Bacteroidetes* amounts and decreased *Firmicutes* amounts in cisplatin-induced pica model of rats, presenting potential property in gut microbiota adjustment against chemotherapy-induced dysbacteriosis [53].

In summary, it is possible that the antioxidative, anti-inflammation, and gut microbiota adjustment effects of gingerols are novel mechanisms in treating CINV. The underlying effects and mechanisms still need further investigations.

## 5. The Potential Mechanisms of Gingerols against CINV Based on Network Pharmacology Prediction

Since gingerols indicate the compounds that all contain 3-methoxy-4-hydroxyphenyl functional group [23], their single ingredients are complex, and their interactions with multiple targets and pathways are varied, it is difficult to investigate the entire mechanism simply using classical pharmacology experiments. With the development of network pharmacology, the connections between ingredients, targets, biological function, and signaling pathways of gingerols against CINV could be clearly demonstrated.

**Figure 3:**
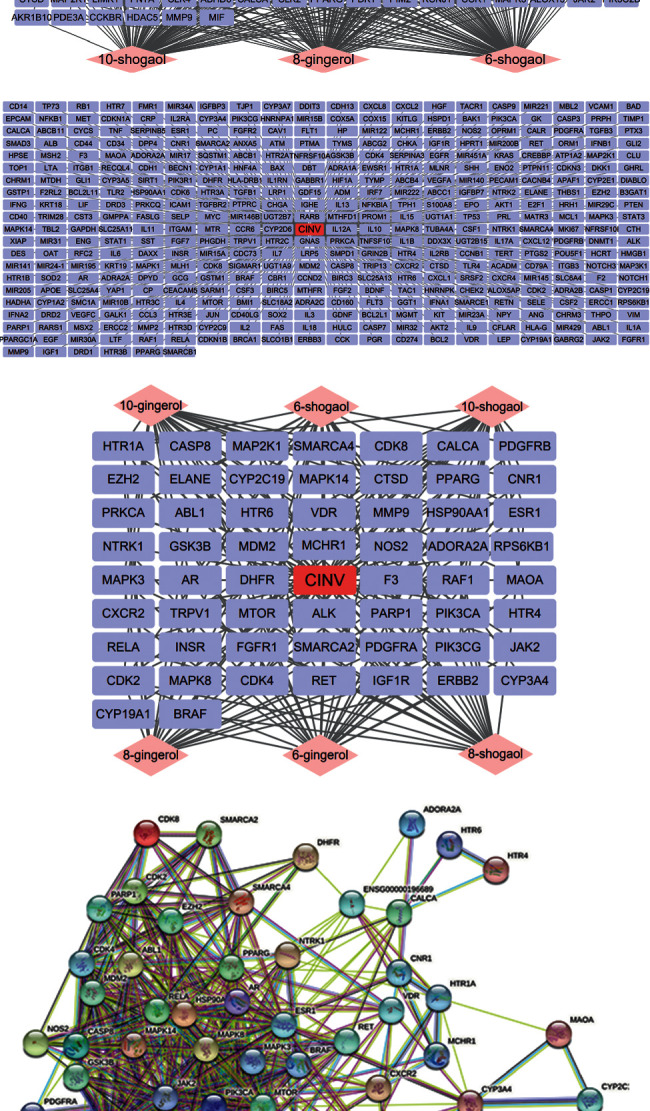
Targets and PPI network of gingerols against CINV. A total of 294 targets of ingredients in gingerols (a) and 405 targets of CINV (b) are screened out. And 57 inserted targets of ingredients and CINV are filtered (c). In these three networks, ingredients are in pink and symbols are in purple. The PPI network of these 57 targets is shown in (d).

The single ingredients of gingerols obtained from the Traditional Chinese Medicine Systems Pharmacology Database and Analysis Platform (TCMSP, http://tcmspw.com/tcmsp.php) are 6-gingerol and 6-shogaol, whose oral bioavailability (OB) ≥ 30% and drug likeness (DL) ≥ 0.14. Other common ingredients including 8-gingerol, 10-gingerol, 8-shogaol, and 10-shogaol are added as well, according to the phytochemical constituents of ginger [12, 22, 23]. Then, the ingredients are imported into the Swiss Target Prediction database (http://www.swisstargetprediction.ch/) to obtain targets. A total of 294 gene targets are obtained and the ingredient-targets network is constructed as shown in Figure 3(a). The DisGeNET database (https://www.disgenet.org/), TTD database (http://db.idrblab.net/ttd/), and DrugBank database (https://www.drugbank.ca/) are utilized to screen out disease targets. There are 405 targets of CINV in total, and the disease network is constructed (Figure 3(b)). The duplicate values are determined to elucidate the common targets of ingredients and CINV; the network of 57 intersected targets is constructed (Figure 3(c)). The details of 57 targets are presented in Table 1. Results suggest that single ingredients like 6-gingerol are able to act on different serotonin receptors and MAO-A, which is consistent with previous reports [10]. The protein-protein interaction (PPI) network is built by STRING database (https://www.string-db.org/) (Figure 3(d)); the top 10 genes according to degree and its relevant effects are shown in Table 2. Through interacting with these genes, the effect of gingerols against CINV may be partly on account of ameliorating cytotoxicity, inflammation, and gastrointestinal dysfunctions induced by chemotherapy agents.

**Figure 4:**
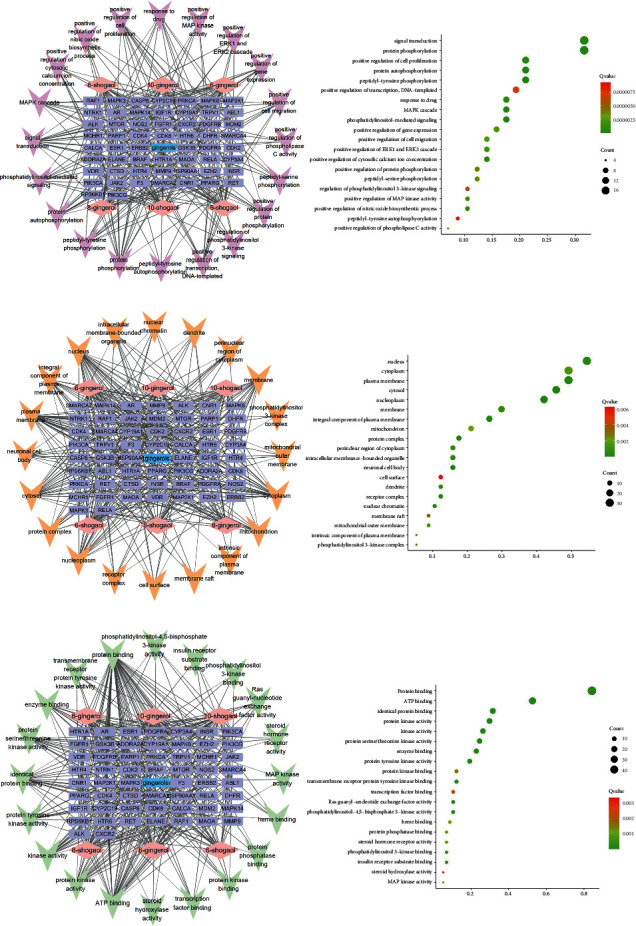
The BP, CC, and MF networks and GO plots of gingerols against CINV. In these three networks, single ingredients of gingerols are in pink and gene symbols in purple. The biological process of BP network (a) is in light pink, the cellular components of CC network (b) in yellow, and the molecular functions of MF (c) network in green. The GO-BP, GO-CC, and GO-MF are presented; red presents higher and green presents lower *p*-value.

**Figure 5:**
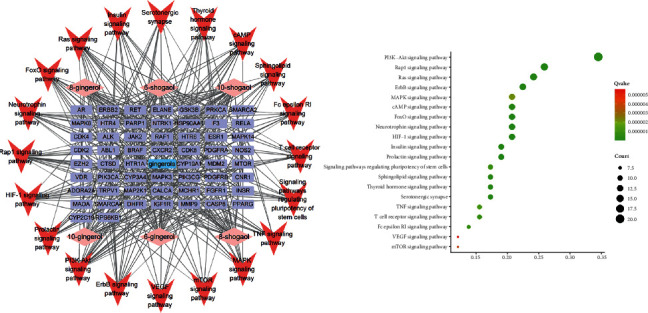
The KEGG pathway network and GO plot of gingerols against CINV. In the network, single ingredients of gingerols are in pink, gene symbols are in purple, and pathway is in red. The GO-KEGG is presented in (b); red presents higher and green presents lower *p*-value.

By using the Database for Annotation, Visualization and Integrated Discovery (DAVID) v 6.8 (https://david.ncifcrf.gov/), the biological process (BP), cellular component (CC), molecular function (MF), and Kyoto Encyclopedia of Genes and Genomes (KEGG) pathways are predicted. There are 235 BP, 37 CC, 39 MF, and 99 KEGG pathways in total. By using the ImageGP online mapping software (http://www.ehbio.com/ImageGP/), GO analyses are performed. Networks and GO enrichment plots of top 20 BP (Figure 4(a)), CC (Figure 4(b)), and MF (Figure 4(c)) according to *p*-value are shown. Also, the network and GO enrichments of top 20 signaling pathways are constructed, after excluding pathways irrelevant to CINV (Figure 5). Interestingly, the KEGG enrichment predicts the PI3K-AKT signaling pathway, which is correlative to the intestinal inflammation [61, 69]. Luettig et al. had proven that 6-shogaol was able to ameliorate intestinal inflammation by affecting PI3K-AKT signaling pathway [70]. Therefore, the alleviation of intestinal damages through PI3K-AKT signaling pathway could be a novel mechanism of gingerols against chemotherapy-induced intestinal inflammation, which might ameliorate CINV. Besides, the results of KEGG prediction also include Rap1 and Ras signaling pathway, all of which interact with downstream ERK/MAPK signaling pathway [71]. Previous study reported that the increased level of ERK contributed to the cell proliferation in intestinal mucosa and accelerated the repair of chemotherapy-induced intestinal damages, thus ameliorating inflammation consequently [72]. Therefore, the effect of gingerols against CINV may also concern with the intestinal injuries repair acceleration via Rap1 and Ras signaling pathway.

By using network pharmacology, the interaction between multiple targets and pathways of gingerols is clearly displayed. The anti-inflammation activity of gingerols through acting on PI3K-AKT signaling pathway, Rap1 signaling pathway, and Ras signaling pathway could be a novel mechanism in preventing CINV. However, although the gingerols isolated from ginger extract might contain all monomers as network pharmacology predicted [22], their potential effects on CINV might be absent since the concentrations of some monomers in gingerols might be below the minimum effect dose. Therefore, future studies focusing on the effect of both monomers in gingerols and gingerols themselves to treat CINV may further identify the underlying antiemetic mechanism.

## 6. Conclusion and Prospect

CINV is still a great challenge in oncotherapy, and the mechanisms of CINV remain incompletely clarified. It is essential to further investigate the underlying mechanisms of CINV and to develop new approaches that have promising effect and few adverse reactions at the same time.

Ginger is a traditional herb that has a promising effect against nausea and vomiting [73]. Gingerols are the major pungent ingredients in ginger, and studies have proven the effect of gingerols in treating CINV. The single ingredients contained in gingerols include 6-, 8-, 10-gingerol, 6-, 8-, 10-shogaol, and others, with 6-gingerol and 6-shogaol being the most abundant. Gingerols distribute widely in the digestive system and could penetrate the blood-brain barrier, which make it a viable approach in treating CINV, a disease closely related to the gastrointestinal tract and brain [74]. The mechanisms of gingerols in ameliorating CINV have not been fully demonstrated yet. Previous studies proved that gingerols effectively prevented CINV via neurotransmitters (including 5-HT, SP, and DA) regulation, gastrointestinal function improvements, gut microbiota adjustment, and anti-inflammation and antioxidative properties.

Through network pharmacology analysis, we predict potential mechanisms of gingerols against CINV. The results concisely exhibit integrated and systematic networks of the interaction between gingerols and disease, demonstrating possible targets and signaling pathways. Network pharmacology carries out novel prospects that gingerols may prevent CINV via reducing inflammation and modulating gastrointestinal function. Future studies may focus on the anti-inflammation property, gastric emptying modulation, and the adjustment of gut microbiota to explore novel mechanisms of gingerols in treating CINV.

## Figures and Tables

**Table 1:** The 57 intersected targets of CINV and gingerols.

Number	Symbol	Targets	Ingredients
1	ABL1	Tyrosine-protein kinase ABL1	8-Gingerol, 10-gingerol, 6-shogaol, 8-shogaol
2	ADORA2A	Adenosine A2a receptor	8-Shogaol, 10-shogaol
3	ALK	ALK tyrosine kinase receptor	6-Gingerol, 6-shogaol, 8-shogaol, 10-shogaol
4	AR	Androgen receptor	6-Gingerol, 8-gingerol, 10-gingerol, 6-shogaol, 8-shogaol, 10-shogaol
5	BRAF	Serine/threonine-protein kinase B-RAF	6-Shogaol, 8-shogaol, 10-shogaol
6	CASP8	Caspase-8	6-Gingerol
7	CDK2	Cyclin-dependent kinase 2/cyclin E1	6-Gingerol, 8-gingerol, 10-gingerol, 6-shogaol, 8-shogaol, 10-shogaol
8	CDK4	Cyclin-dependent kinase 4	6-Shogaol, 10-shogaol
9	CDK8	Cyclin-dependent kinase 8	6-Gingerol, 8-gingerol, 10-shogaol
10	CNR1	Cannabinoid receptor 1	6-Gingerol, 8-gingerol, 10-gingerol, 10-shogaol
11	CTSD	Cathepsin D	10-Gingerol
12	CXCR2	C-X-C chemokine receptor type 2	6-Gingerol, 10-gingerol
13	CYP19A1	Cytochrome P450 19A1	6-Gingerol
14	CALCA	Calcitonin gene-related peptide 1	10-Shogaol
15	CYP2C19	Cytochrome P450 2C19	6-Shogaol
16	CYP3A4	Cytochrome P450 3A4	6-Shogaol
17	DHFR	Dihydrofolate reductase	8-Shogaol, 10-shogaol
18	ELANE	Neutrophil elastase	6-Shogaol, 8-shogaol
19	ERBB2	Receptor protein-tyrosine kinase ERBB-2	6-Gingerol, 8-gingerol, 10-gingerol, 8-shogaol
20	ESR1	Estrogen receptor	6-Gingerol, 8-gingerol, 10-gingerol, 6-shogaol, 8-shogaol, 10-shogaol
21	F3	Coagulation factor VII/tissue factor	10-Shogaol
22	EZH2	Histone-lysine N-methyltransferase EZH2	8-Gingerol
23	FGFR1	Fibroblast growth factor receptor 1	6-Shogaol, 8-shogaol
24	GSK3B	Glycogen synthase kinase-3 beta	6-Gingerol
25	HSP90AA1	Heat shock protein HSP 90-alpha	6-Gingerol, 10-gingerol
26	HTR4	Serotonin 4 (5-HT4) receptor	10-Shogaol
27	HTR1A	Serotonin 1a (5-HT1a) receptor	6-Gingerol, 8-gingerol, 10-gingerol, 6-shogaol, 8-shogaol, 10-shogaol
28	HTR6	Serotonin 6 (5-HT6) receptor	8-Shogaol
29	IGF1R	Insulin-like growth factor I receptor	6-Gingerol, 8-gingerol, 10-gingerol, 10-shogaol
30	INSR	Insulin receptor	8-Gingerol, 10-gingerol
31	JAK2	Tyrosine-protein kinase JAK2	6-Gingerol, 10-gingerol
32	MAO-A	Monoamine oxidase A	6-Gingerol, 10-gingerol
33	MAP2K1	Dual specificity mitogen-activated protein kinase 1	6-Gingerol, 8-gingerol, 10-gingerol
34	VDR	Vitamin D3 receptor	10-Shogaol
35	RPS6KB1	Ribosomal protein S6 kinase beta-1	10-Shogaol
36	MAPK14	Mitogen-activated protein kinase 14	6-Shogaol, 8-shogaol
37	MAPK3	Mitogen-activated protein kinase 3	6-Gingerol, 8-gingerol, 10-gingerol, 8-shogaol
38	MCHR1	Melanin-concentrating hormone receptor 1	10-Gingerol, 10-shogaol
39	MDM2	E3 ubiquitin-protein ligase Mdm2	8-Gingerol, 10-gingerol
40	MMP9	Matrix metalloproteinase-9	6-Shogaol, 8-shogaol, 10-shogaol
41	MTOR	Serine/threonine-protein kinase mTOR	6-Gingerol, 8-gingerol, 10-gingerol, 6-shogaol, 8-shogaol, 10-shogaol
42	NOS2	Nitric oxide synthase	6-Gingerol
43	NTRK1	High-affinity nerve growth factor receptor	6-Gingerol
44	MAPK8	Mitogen-activated protein kinase 8	10-Shogaol
45	PARP1	Poly[ADP-ribose] polymerase-1	6-Gingerol, 8-gingerol, 6-shogaol, 8-shogaol, 10-shogaol
46	PDGFRA	Platelet-derived growth factor receptor alpha	6-Shogaol, 8-shogaol
47	PDGFRB	Platelet-derived growth factor receptor beta	6-Shogaol, 10-shogaol
48	PIK3CA	Phosphatidylinositol 4,5-bisphosphate 3-kinase catalytic subunit alpha isoform	6-Gingerol, 8-gingerol, 10-gingerol, 6-shogaol, 8-shogaol, 10-shogaol
49	PIK3CG	Phosphatidylinositol 4,5-bisphosphate 3-kinase catalytic subunit gamma isoform	6-Gingerol, 8-gingerol, 10-gingerol, 6-shogaol, 8-shogaol, 10-shogaol
50	PPARG	Peroxisome proliferator-activated receptor gamma	6-Gingerol, 8-gingerol, 10-gingerol
51	PRKCA	PRKCA-binding protein	6-Shogaol
52	RAF1	RAF protooncogene serine/threonine-protein kinase	8-Shogaol, 10-shogaol
53	RELA	Nuclear factor NF-kappa-B p65 subunit	8-Shogaol
54	RET	Protooncogene tyrosine-protein kinase receptor RET	6-Gingerol, 8-gingerol, 10-gingerol
55	SMARCA2	Probable global transcription activator SNF2L2	6-Shogaol
56	SMARCA4	Transcription activator BRG1	6-Shogaol
57	TRPV1	Transient receptor potential cation channel subfamily V member 1	8-Shogaol, 10-shogaol

**Table 2:** The top 10 genes and their relevant effects.

Gene name	Degree	Betweenness centrality	Closeness centrality	Relevant effects
MAPK3	38	0.133309434	0.756756757	Relates to the gastric emptying process [54]
MTOR	38	0.104807106	0.736842105	Regulates cell proliferation, survival, motility, apoptosis, and concerned with the expression of appetite regulating peptides [55, 56]
ESR1	34	0.06775269	0.717948718	Encodes estrogen *α*/*β* that involve in the regulation of feeding behavior [57]
HSP90AA1	31	0.032249982	0.666666667	Mediates cell autophagy [58, 59]
PIK3CA	31	0.047945105	0.666666667	Modulates cell apoptosis and autophagy via PI3K-AKT signaling pathway, which ameliorates intestinal cytotoxicity of chemotherapy agents [60, 61]
MDM2	30	0.030363847	0.658823529	The inhibition of MDM2 via Notch/hes1 or NF-*κ*B pathway improves chemotherapy agents-induced cytotoxicity [62, 63]
MAPK8	28	0.03301698	0.643678161	Intensifies the inflammatory and apoptotic of intestinal epithelial cells [64, 65]
ERBB2	27	0.019357326	0.658823529	Associates with DNA repair and the cytotoxicity of chemotherapy agents [66]
AR	27	0.03325207	0.636363636	Ameliorates early mortality through regulating gut microbiota [67]
JAK2	26	0.018936076	0.629213483	Mediates leptin level and regulates food intake [68]
